# Spatiotemporal profiling of the bovine oviduct fluid proteome around the time of ovulation

**DOI:** 10.1038/s41598-022-07929-3

**Published:** 2022-03-09

**Authors:** Coline Mahé, Régis Lavigne, Emmanuelle Com, Charles Pineau, Yann Locatelli, Aleksandra Maria Zlotkowska, Carmen Almiñana, Guillaume Tsikis, Pascal Mermillod, Jennifer Schoen, Marie Saint-Dizier

**Affiliations:** 1 CNRS, IFCE, INRAE, Université de Tours, PRC, 37380 Nouzilly, France; 2 Inserm, EHESP, Irset (Institut de Recherche en santé, environnement et travail) – UMR-S 1085, Univ Rennes, 35000 Rennes, France; 3 Protim, Biosit – UMS 3480 CNRS, US 018 Inserm, Univ Rennes, 35000 Rennes, France; 4 Laboratoire de la Réserve Zoologique de la Haute Touche, MNHN, Obterre, France; 5 Institute of Reproductive Biology, Leibniz Institute for Farm Animal Biology, FBN, Dummerstorf, Germany; 6 Functional Genomics Group, Institute of Veterinary Anatomy, Vetsuisse Faculty Zurich, University of Zurich, 8315 Lindau, Switzerland; 7 Tours University, Faculty of Sciences and Techniques, Tours, France; 8 Present Address: Department of Reproduction Biology, Leibniz Institute for Zoo and Wildlife Research (IZW), Berlin, Germany

**Keywords:** Developmental biology, Molecular biology

## Abstract

Understanding the composition of the oviduct fluid (OF) is crucial to better comprehend the microenvironment in which sperm capacitation, fertilization and early embryo development take place. Therefore, our aim was to determine the spatiotemporal changes in the OF proteome according to the anatomical region of the oviduct (ampulla *vs.* isthmus), the proximity of the ovulating ovary (ipsilateral *vs*. contralateral side) and the peri-ovulatory stage (pre-ovulatory or Pre-ov *vs*. post-ovulatory or Post-ov). Oviducts from adult cyclic cows were collected at a local slaughterhouse and pools of OF were analyzed by nanoLC-MS/MS and label-free protein quantification (n = 32 OF pools for all region × stage × side conditions). A total of 3760 proteins were identified in the OF, of which 65% were predicted to be potentially secreted. The oviduct region was the major source of variation in protein abundance, followed by the proximity of the ovulating ovary and finally the peri-ovulatory stage. Differentially abundant proteins between regions, stages and sides were involved in a broad variety of biological functions, including protein binding, response to stress, cell-to-cell adhesion, calcium homeostasis and the immune system. This work highlights the dynamic regulation of oviduct secretions and provides new protein candidates for interactions between the maternal environment, the gametes and the early embryo.

## Introduction

In vivo, pregnancy establishment requires a sequence of events in the different segments of the oviduct around the time of ovulation. After migration through the uterus, sperm reach the isthmus (i.e., the caudal part of the oviduct) and bind to oviduct epithelial cells (OECs) to form a sperm reservoir until ovulation time when they are gradually released toward the ampulla^1,2^. From the isthmus to the ampulla, spermatozoa are progressively capacitated and acquire the ability to fertilize oocytes^3^. In the immediate post-ovulatory period, the oocyte and surrounding cumulus cells go through the ampulla where fertilization takes place^4^. In the 3–5 days following ovulation, cell divisions of the zygote start in the isthmus and the early embryo finally enters the uterus^5^.

Under physiological conditions, the oviduct fluid (OF) offers an optimal environment for these early reproductive events. The OF is a complex mixture of small metabolites, lipids, glycans and proteins originating from secretions of the luminal epithelium, selective ultrafiltrate from the circulating plasma and putative inputs from the follicular fluid^6^. Previous work from our group showed that the steroidomic^7^, metabolomic^8^ and proteomic^9^ composition of the bovine OF varied throughout the estrous cycle and according to the proximity of the Pre-ov follicle (POF) or corpus luteum (CL). Furthermore, variations in gene expression have been reported between ampulla and isthmus^10–13^. However, whether these variations in gene expression are translated into variations in OF protein abundance between ampulla and isthmus is currently not known. Recently, a small set of OF proteins were reported to vary in abundance between cyclic and pregnant cows on day 3 post-estrus, with slight differences between the ampulla and isthmus^14^. Region-related differences in oviductal secretions were reported in vitro: effects of OF-derived extracellular vesicles (EVs)^15^ and OF proteins^16^ on sperm viability and capacitation differed between ampulla and isthmus. Moreover, the quality of embryos developed in vitro with bovine OF-derived EVs^17^ or ovine OECs^18^ differed depending on the ampullary or isthmic origin of the EVs or OECs.

The spatiotemporal variation in OF proteome remains unclear. We hypothesize that the OF proteome is monitored by a complex set of factors: the anatomical region of the oviduct, the proximity of POF/CL and the stage of cycle. A dynamic variation between the ampulla and isthmus in the oviduct ipsilateral to ovulation, but also between the Pre-ov and Post-ov stages are expected, in response to the specific requirements of gametes and embryos. Therefore, the objective of this study was to determine the effect on the OF proteome of (1) the region (isthmus *vs*. ampulla), (2) the proximity of the ovulating ovary (ipsilateral *vs*. contralateral side) and (3) the peri-ovulatory stage (Pre-ov *vs*. Post-ov) using nanoLC-MS/MS analysis.

## Results

### Proteins identified in the bovine OF and predicted secretory pathways

**Figure 1:**
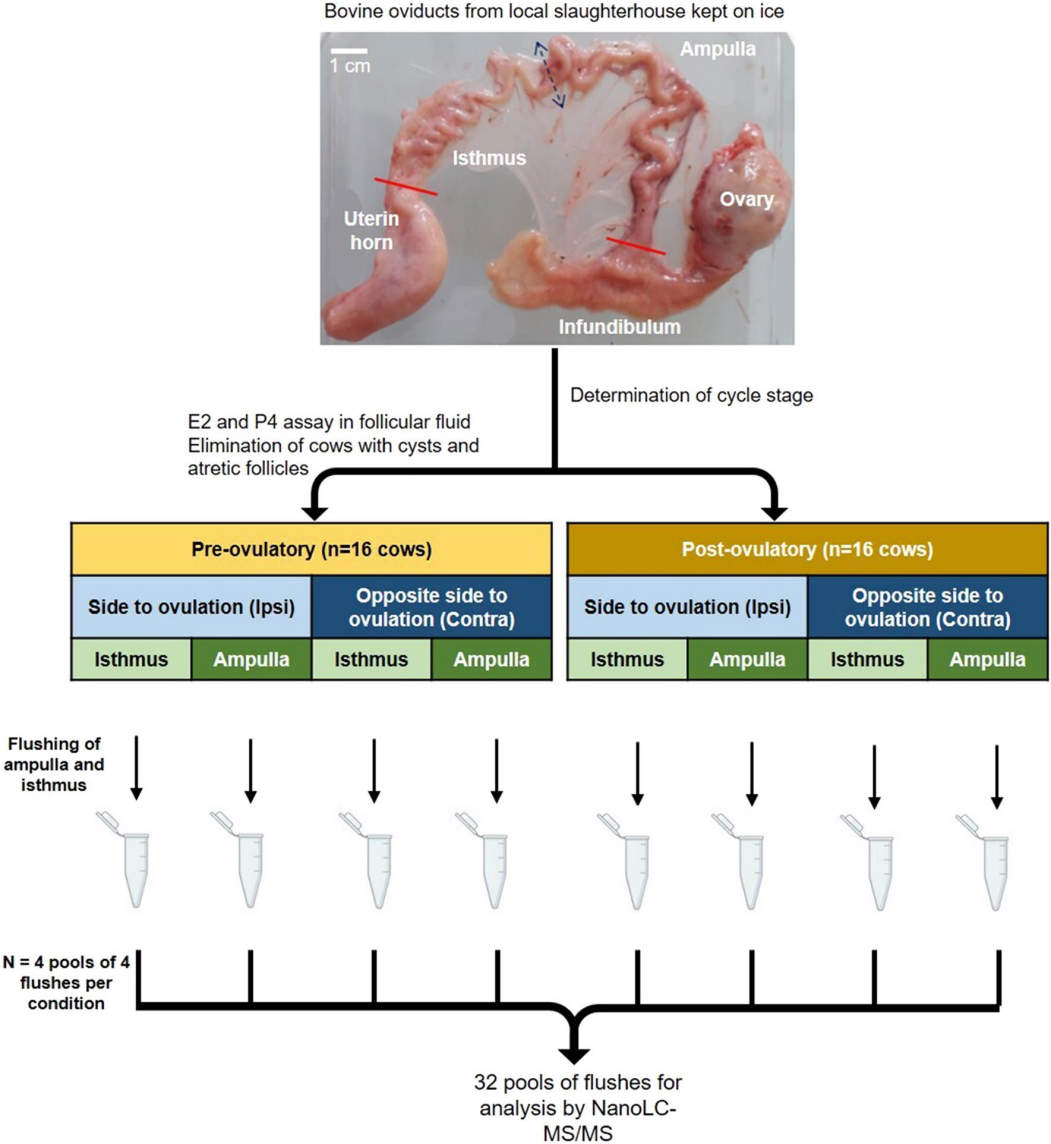
Experimental design of bovine oviduct fluid collection. E2: 17β-estradiol; P4: progesterone. See “Methods” section for further details.

Thirty pools of OF were made (4 cows per pool, 8 region × stage × side conditions) and analyzed by nanoLC-MS/MS (see Fig. 1). A total of 3760 proteins were identified in the bovine OF (Supplementary Data S1). To determine if some proteins were specific to one region, stage or side, the overlap between conditions was evaluated. Overall, 77% (2894/3760) of proteins were shared between conditions. Of the remaining 23%, only 34 proteins were quantified with more than 5 normalized weighted spectra (NWS, mean values) in one specific region, side or stage (see Supplementary Data S1).

To determine the potential pathways by which proteins were exported into the OF, peptide signal sequences were identified and unconventional secretion pathways were predicted using the Outcyte 1.0 and SignalP 5.0 server on the list of protein gene names. Overall, 37% (1373/3738) of the proteins identified were shown as potentially secreted proteins. Of those, 10% (360/3738) contained a signal peptide and were predicted to be classically secreted while 27% (1013/3738) were predicted to be potentially secreted via unconventional pathways (see the prediction scores and cleavage site positions in Supplementary Data S1). In addition, 41% (1551/3738) of the proteins identified in the bovine OF were previously reported in EVs derived from oviduct epithelial cells (see the list of proteins and origin in Supplementary Data S1)^19–21^. Of the 1551 proteins potentially originated in oEVs, 498 (13%) were previously predicted as potentially secreted by the Outcyte and SignalP tools. By merging the three lists, a total of 2426 proteins (65%) were predicted to be secreted by classical or non-classical (including EVs) pathways.

### Spatiotemporal profiling of the OF proteome and functional analysis

**Figure 2:**
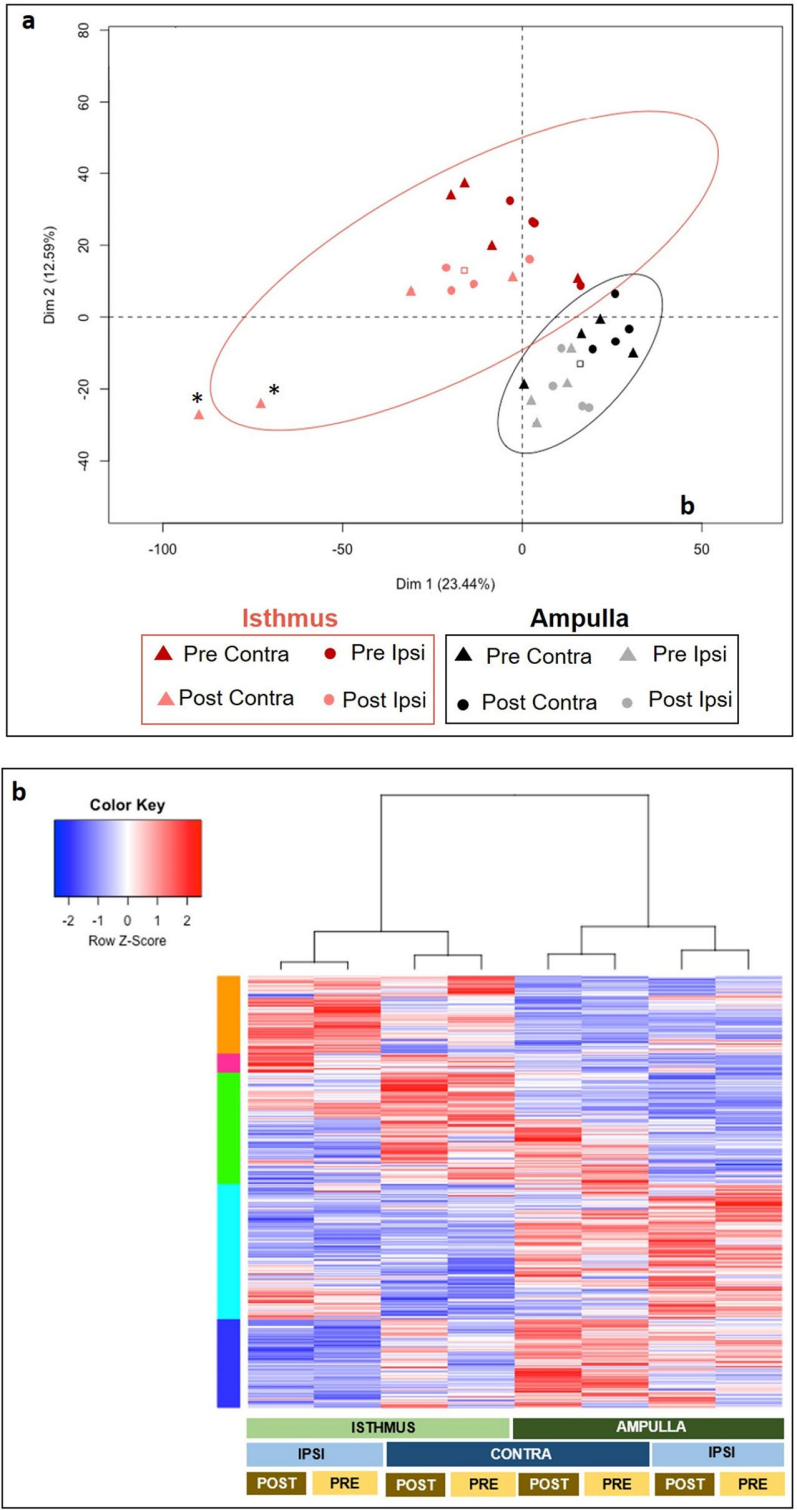
Principal component analysis of all samples (**a**) and heatmap of the 1491 differentially abundant proteins (**b**). (**a**) Principal component analysis of the 32 OF samples showing a separation between regions of the oviduct (red, isthmus; black, ampulla). Each spot represents one biological sample in a given region × stage × side condition (see legends for corresponding form and color). Each ellipse encloses 93–100% of samples for each region. The square in each ellipse represents the mean of data for a given region. Two outsiders in the Pre-ov ipsilateral isthmus group (asterisks) were discarded. (**b**) Heatmap and hierarchical clustering of the 1491 differentially abundant proteins (DAPs; ANOVA p-value ≤ 0.05) identified across regions, stages and sides. Each line corresponds to one protein. For a given protein, blue lines represent higher abundance while red lines represent lower abundance compared to other conditions. White lines represent the median abundance values. Five clusters of proteins were identified by unsupervised hierarchical clustering (vertical multicolored bars on the left). Light and dark blue bars indicate clusters with higher abundance in the isthmus than in the ampulla while orange bar indicates one cluster displaying higher abundance in the ampulla than in the isthmus. The proximity between regions, stages and side of ovulation are shown by the hierarchical tree on top.

To obtain an overview of proteomic data, a principal component analysis (PCA) of proteins quantified with min 2 normalized weighted spectra (NWS) in at least one condition was carried out (3009 proteins). PCA showed a clear separation between the ampullary and isthmic samples (Fig. 2a). Two samples in the Pre-ov ipsilateral isthmus group had to be discarded from further analysis because they had a low MS signal resulting in 50% less proteins identified than in all other samples.

**Figure 3:**
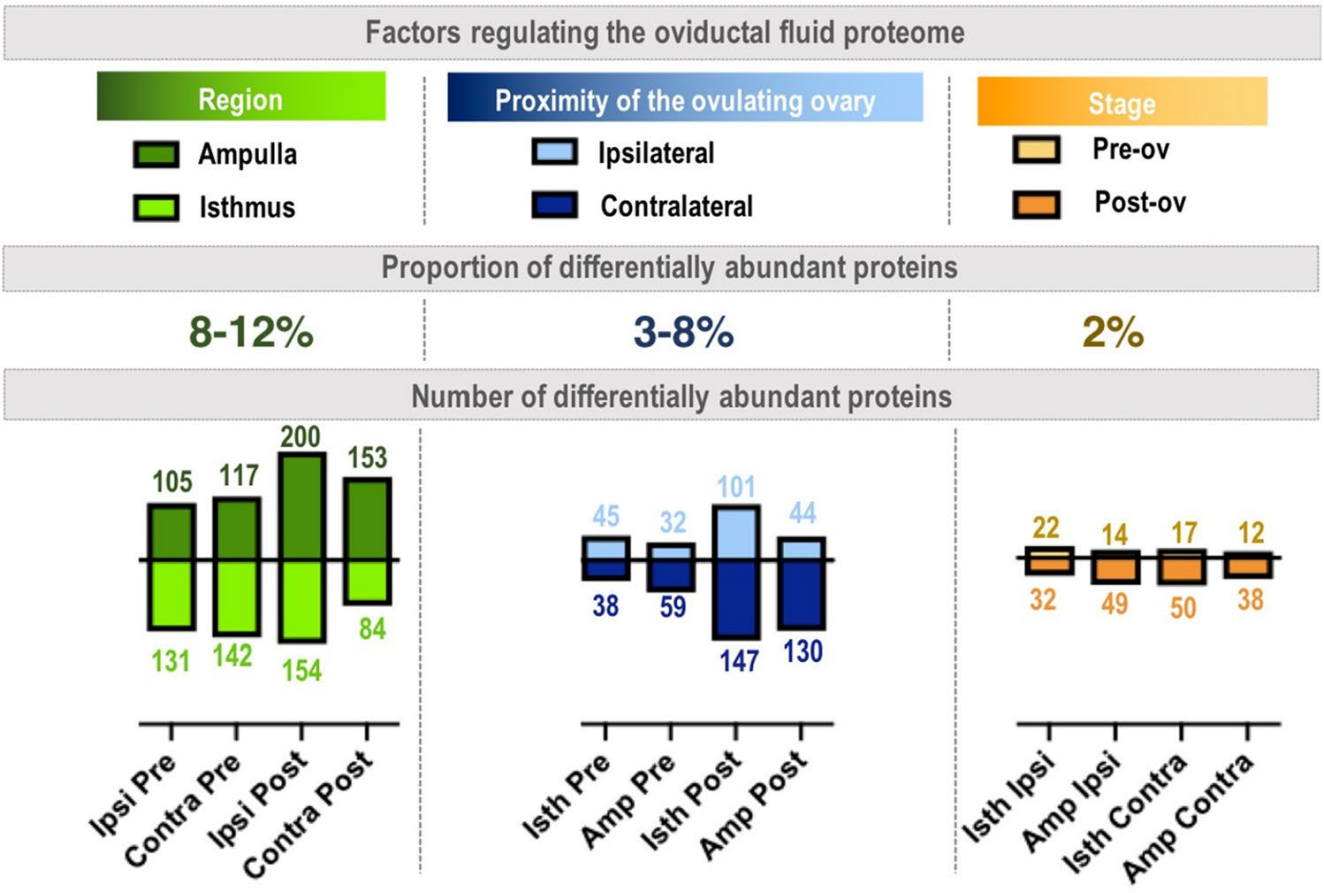
Numbers and proportions of differentially abundant proteins according to the region, proximity of ovulating ovary and peri-ovulatory stage. Proteins were considered as differentially abundant with a t-test p-value ≤ 0.05 and a fold-change ratio ≥ 2 or ≤ 0.5. Each bar represents the number of overabundant proteins (with a min mean value of 2 NWS) in each region × stage × side conditions.

**Figure 4:**
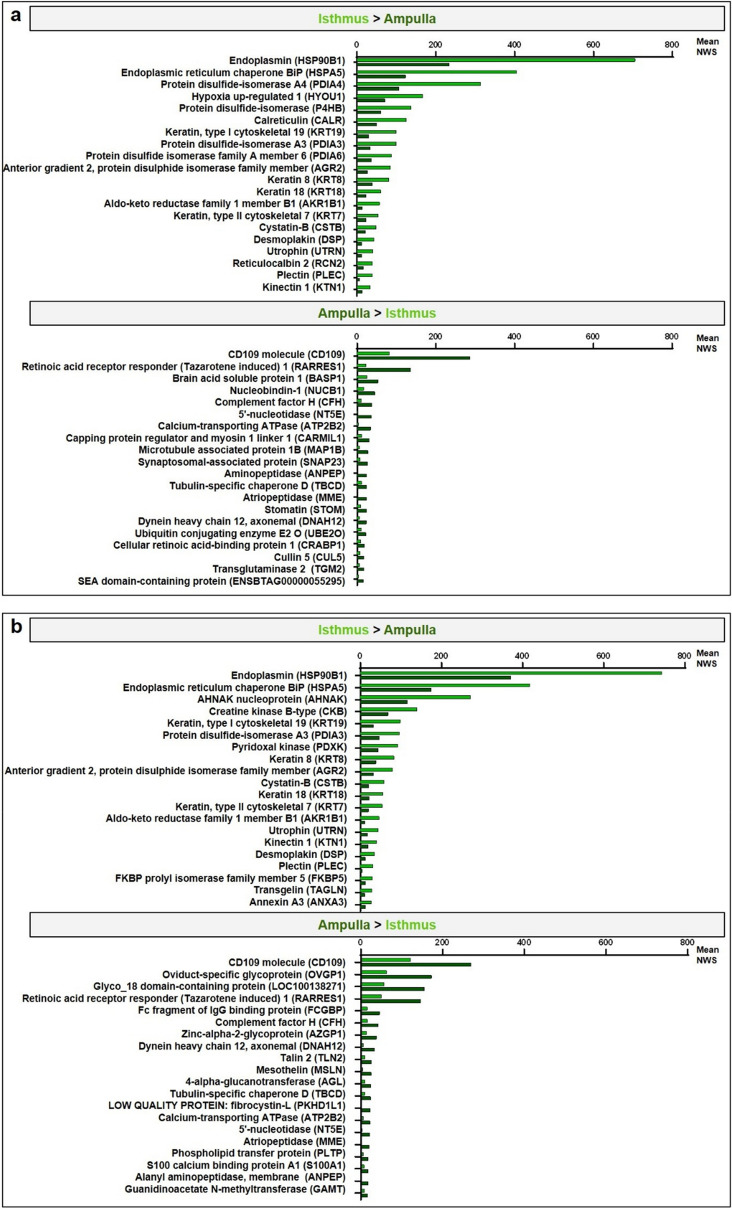
Mean quantitative values of the top-20 differentially abundant proteins between regions in the ipsilateral oviduct at pre-ovulatory (**a**) and post-ovulatory (**b**) stages.

**Figure 5:**
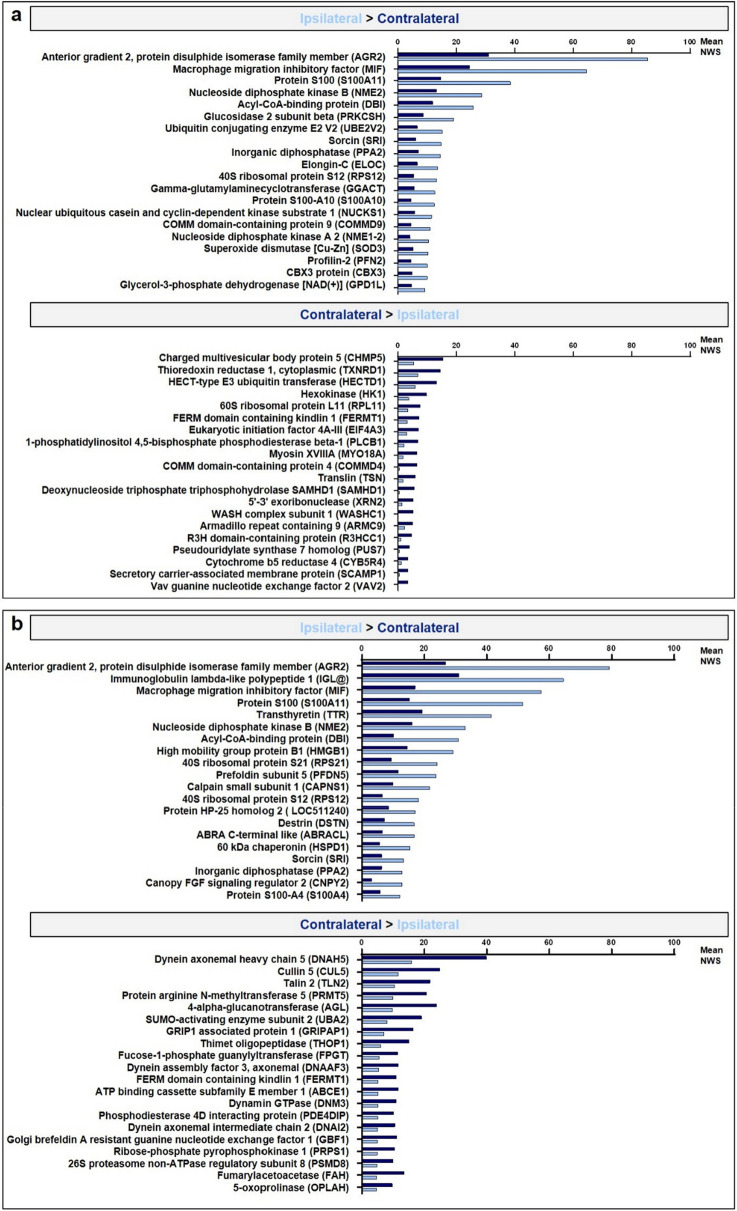
Mean quantitative values of the top-20 differentially abundant proteins according to the proximity of the ovulating ovary in the isthmus at pre-ovulatory (**a**) and at post-ovulatory (**b**) stages.

**Figure 6:**
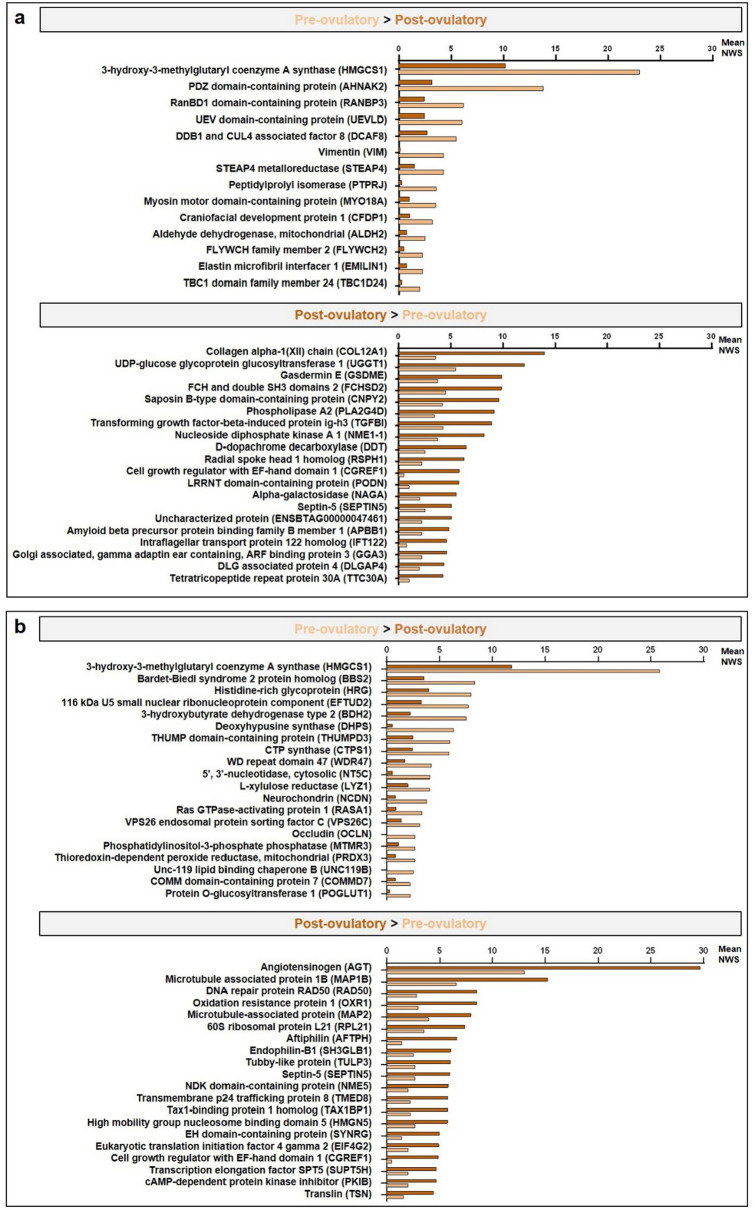
Mean quantitative values of top-20 differentially abundant proteins between stages in the ipsilateral ampulla (**a**) and isthmus (**b**).

To evaluate more specifically which factors among the region, the peri-ovulatory stage and the proximity of the ovulating ovary most affected the OF proteome, a hierarchical clustering of differentially abundant proteins (DAPs, ANOVA p-value ≤ 0.05) was performed. The heatmap representation of this analysis confirmed that the main differences in protein abundance were between the anatomical regions (ampulla *vs*. isthmus), followed by the proximity of the ovulating ovary (ipsilateral *vs*. contralateral) and finally the stage around ovulation time (Pre-ov *vs*. Post-ov) (Fig. 2b). Based on these findings, statistical analyses comparing ampulla *vs*. isthmus, ipsilateral *vs*. contralateral and Pre-ov *vs*. Post-ov datasets were performed. Overall, pair-wise comparisons (t-test p-value ≤ 0.05) considering a fold-change ratio ≥ 2 evidenced highest numbers of DAPs when comparing ampulla and isthmus (236–354 DAPs, *i.e.,* 8–12% of quantified proteins), followed by those between ipsi- and contralateral sides (83–248 DAPs, i.e. 3–8% of quantified proteins) and finally between the Pre-ov and Post-ov stages (50–67 DAPs, i.e. 2% of quantified proteins; see Fig. 3). Interestingly, the post-ovulatory isthmus, *i.e.,* the place and time of early embryo development, displayed the highest number of DAPs when comparing the ipsilateral with contralateral oviducts. Top-20 DAPs between regions, sides of ovulation and stages are shown in Figs. 4, 5 and 6 respectively (see Supplementary Datas S2–4 for all DAPs with accession numbers, molecular weight, p-value and fold-changes).

**Figure 7:**
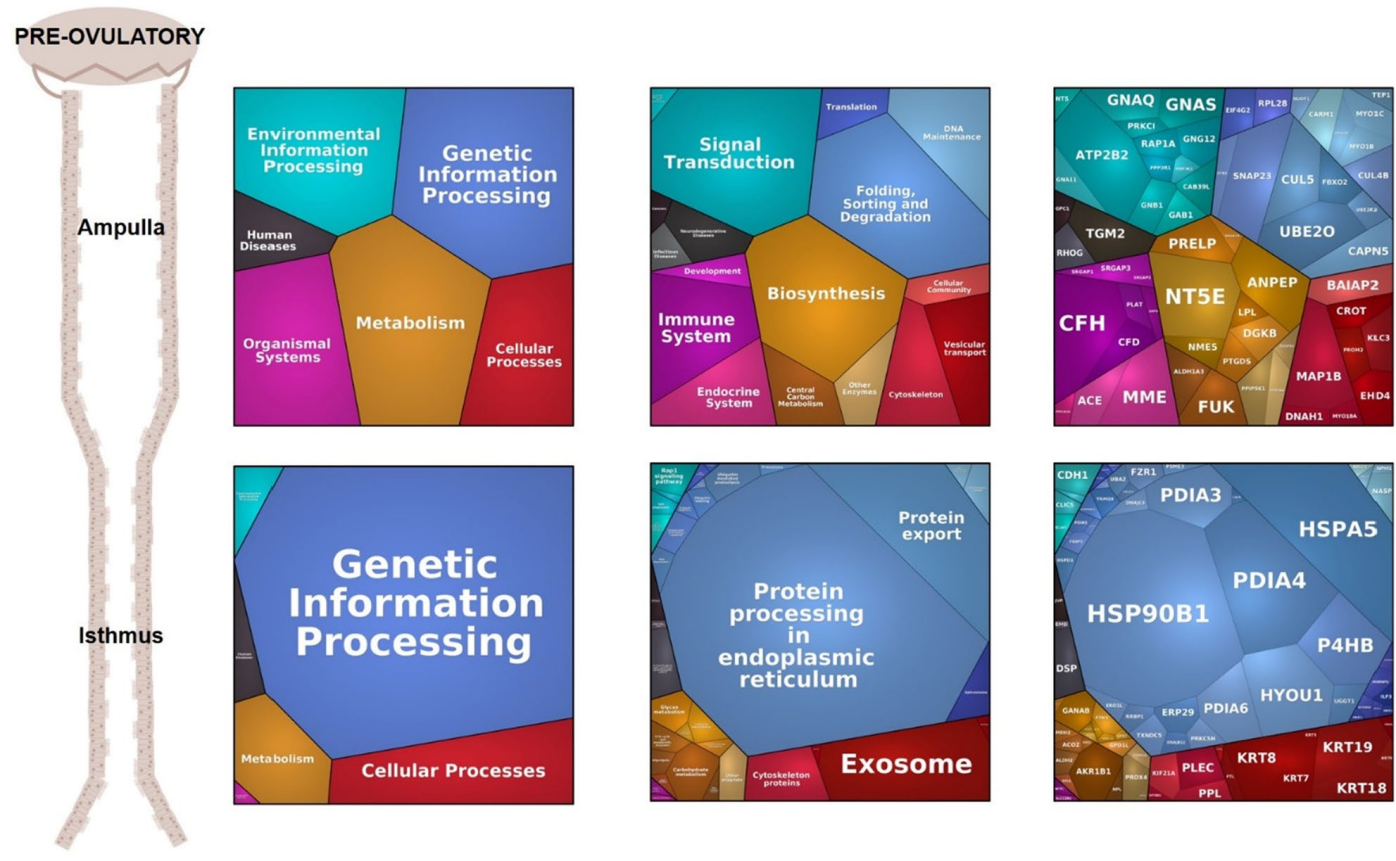
Proteomaps of overabundant proteins in the ipsilateral ampulla and isthmus at Pre-ov stage. Proteomaps were generated using the KEGG (Kyoto Encyclopedia of Genes and Genomes) Pathway gene classification. Functional categories (left and middle panels) and proteins (right panels) are shown by polygons. Areas of polygons illustrate protein normalized abundance in each region. Functions and proteins linked are organized in common regions and coded using similar colors.

**Figure 8:**
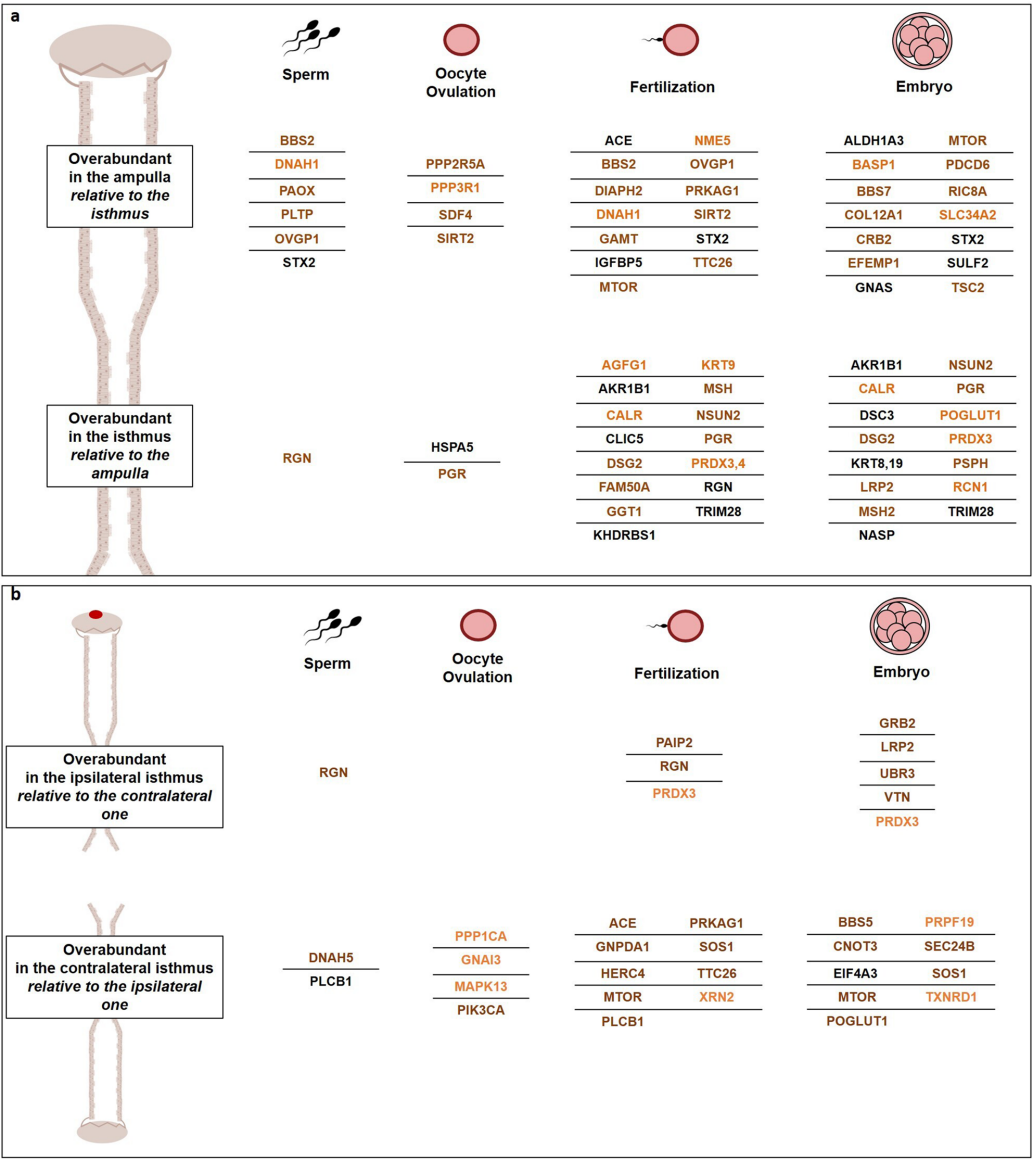
Potential reproductive targets of OF proteins differentially abundant according to the (**a**) ipsilateral regions and (**b**) proximity of the ovulating ovary in the ipsilateral isthmus at both peri-ovulatory stages. Colors indicate the stage at which the protein is overabundant. Orange, overabundant at pre-ovulatory; brown, overabundant at post-ovulatory; black, overabundant at both stages.

To evaluate the functions underlying those variations, an enrichment analysis of genes corresponding to DAPs was undertaken in the Database for Annotation, Visualization and Integrated Discovery (DAVID). This analysis for the effect of the region and stage was performed only on samples from the ipsilateral side. Analysis of the gene ontology (GO) terms associated with DAPs between regions of the oviduct indicated that 67 and 68 biological processes (BP) and molecular functions (MF) were overrepresented (p-value ≤ 0.05) at Pre-ov and Post-ov stages, respectively (Supplementary Data S2). Of those, 21 were shared between stages, including protein binding, cell–cell adhesion, protein folding, calcium ion binding and carbohydrate metabolic process as the most significant at both Pre- and Post-ov (in yellow in Supplementary Data S2). BP/MF overrepresented only at Pre-ov included protein disulfide isomerase activity, response to endoplasmic reticulum stress, redox homeostasis, negative regulation of apoptotic process, and microtubule motor activity (in green in Supplementary Data S2). BP/MF overrepresented only at Post-ov included calcium-dependent protein binding, calmodulin binding and cellular response to catecholamine stimulus (in blue in Supplementary Data S2). To further decipher the role of DAPs, Proteomaps were carried out on overabundant proteins in each region of the ipsilateral oviduct. At Pre-ov, proteins overabundant in the isthmus were mainly involved in protein processing in endoplasmic reticulum and protein export, while proteins overabundant in the ampulla were more specifically involved in the immune and endocrine systems in addition to the isthmic pathways (Fig. 7). Proteomaps generated at post-ovulatory stages displayed globally the same pathways (Supplementary Fig. S2). To point out proteins potentially involved in the reproductive events taking place in the oviduct, proteins overabundant in the ipsilateral isthmus or ampulla were further analyzed through the Metascape^22^ membership tool. Proteins associated with the membership terms ‘sperm’, ‘oocyte/ovulation’, ‘fertilization’ and ‘embryo’ are shown in Fig. 8 (see the exhaustive list of GO terms in Membership analysis Metascape Supplementary Data S2).

Regarding the DAPs between ipsilateral and contralateral oviducts, analysis of the GO terms associated with DAPs between ipsilateral and contralateral oviducts evidenced protein binding as the only enriched MF shared between conditions (Supplementary Data S3). In the ampulla, overrepresented BP/MF included protein binding, endosomal transport, protein transport and multivesicular body assembly among the most significant GO terms at both stages. In the isthmus, the highest number (61) of specifically enriched GO terms was evidenced at Post-ov including mucus secretion, epidermal growth factor (EGF) receptor signaling pathway, ERBB2 signaling pathway and 1-phosphatidylinositol (PI)-3-kinase activity as the most significant ones. In addition, a number of DAPs between sides were associated with the membership terms related to early reproductive events (Fig. 8b and Supplementary Data S3).

Regarding the DAPs between Pre-ov and Post-ov stages in the ipsilateral oviduct, analysis of the GO terms associated with DAPs between Pre-ov and Post-ov samples in the ipsilateral oviduct evidenced 6 and 5 overrepresented BP/MF in the ampulla and isthmus, respectively, of which only protein binding was shared (Supplementary Data S4). BP overrepresented exclusively in the ampulla included regulation of cilium assembly, intraciliary transport and negative regulation of cell proliferation while artery smooth muscle contraction and vasodilatation were overrepresented only in the isthmus (see Supplementary Data S4). The DAPs associated with membership terms related to oviduct events are shown in Supplementary Data S4.

## Discussion

The present study provides an extensive analysis of the bovine OF proteome considering three factors of variation, i.e., the anatomical oviduct region, the peri-ovulatory stage and the proximity of the ovulating ovary. The main findings of this study are that (i) 8 to 12% of the proteins quantified varied in abundance between ampulla and isthmus, in accordance with our hypothesis that each segment of the oviduct may respond to specific requirements for the gametes and embryos in case of pregnancy; (ii) the region of the oviduct was more determining than the proximity of the ovulating ovary and the peri-ovulatory stage on protein abundance; (iii) OF proteins varying in abundance are involved in a broad variety of biological functions, including protein binding, response to stress, calcium homeostasis, and lipid and carbohydrate metabolism, which may play key roles in gamete/embryo-oviduct interactions.

Transport of oviducts were carried out on ice, bringing contractions and any chemical reactions to a halt and with limited time after animal death. The process of OF collection was conducted at 4 °C and the quality and homogeneity of protein samples were checked by electrophoresis on SDS-PAGE, however, we cannot exclude minor protein modifications due to post-mortem collection. Leese et al. in 2008 recommended that 1–2 min post-mortem was the optimal time for sample collection in laboratory animals but quite difficult with large animals such as cattle^23^. Various methods of collection of the oviduct fluid were used over the past 50 years^23^. In the late 70s, micro-volume of OF were collected by aspiration under general anesthesia in mice^24,25^. To retrieve larger volume of fluid, the OF was later collected by oviduct cannulation through the infundibulum for several weeks in cattle^26,27^. However, alterations of fluid composition by inflammation and flow disruption have pushed towards a faster OF collection within hours under general anesthesia^28^. In the 90s, OF were also collected ex vivo during blood perfusion of the genital tract after hysterectomy in women^29^ and in small laboratory animals like rabbits^30^. However, due to ethical, technical and economic barriers in large animals such as cattle, the post-mortem recovery of oviducts at the slaughterhouse with time periods of 1 to 4 h between animal death and OF collection is preferably used^9,13,14,31,32^. One benefit of post-mortem collection is the absence of contamination with inflammatory products and less hemoglobin than observed after oviduct cannulation^33^.

More than 3700 proteins were identified in the bovine OF, which is the most comprehensive OF proteome published so far in mammals^9,33–36^. From the total of protein identified in our study, 37% were predicted as potentially secreted and 10% contained a signal peptide. These proportions of secreted proteins are comparable to those previously reported in the OF in cattle^34,35^ and sheep^37^. It is unclear how intracellular proteins could be exported in high proportions to the oviductal lumen. Considering the very low volume of OF in both ampulla (< 50 µL) and isthmus (< 10 µL) and to avoid cell damage, the samples were collected by gentle flushing. Centrifugations were then carried out to eliminate cell debris. The high rate of renewal of the oviduct epithelium during estrus^38,39^ may lead to intracellular release of proteins into the OF. Moreover, and in the light of our results, it is very likely that intracellular proteins could be derived from apocrine or non-canonical secretory pathways, as it has been shown for annexins (ANXAs)^19,40^. The number of proteins previously identified in OF-derived EVs were higher in cats and pigs (1149 and 1704 proteins) than in cattle (316 proteins) so all three species were considered for the analysis and in accordance, a high proportion of intracellular proteins were previously identified in EVs derived from OF^19–21^.

The most abundant proteins quantified in the OF included several HSPs (HSP90B1, HSP90AA1, HSP90AB1, HSPA8, HSPA5 also known as GRP78), CD109 molecule, complement C3, myosins (MYH9, MYH14) and the oviduct-specific glycoprotein or oviductin (OVGP1), as previously reported^9,33,35^. The majority (77%) of proteins quantified were detected in all conditions. The remaining 23% had on average low levels of detection (NWS < 5), suggesting that these proteins could have been detected with a higher sensitivity. Although, little attention has been paid to this small set of proteins, we should not rule out that they may have an important role in the oviduct. The use of mass spectrometer with higher sensitivity could determine whether these proteins were only present in a specific segment of the oviduct or stage, although in small amounts.

The region-related changes in the OF proteome are presented for the first time, providing valuable information on the specific requirements of gametes and embryos. Furthermore, the region was the greatest source of variation in the oviduct proteome. These results are in agreement with previous transcriptomic data evidencing differentially expressed genes between the ampulla and isthmus in cattle^10–13^. Also in accordance, the abundance of specific proteins was differently regulated in each region after insemination in rabbits^41^ and between pregnant and cyclic cows^14^. Differences in the vascularization^42^ and in the proportion of ciliated cells^39^ between isthmus and ampulla could explain this variation in OF proteome. It is well known that sex steroid hormones are major regulators of oviduct physiology and secretory activity^6^. To our knowledge, the region-specific concentrations of progesterone and estradiol in the oviduct have not been reported to date. However, the expression of progesterone receptors was reported to vary according to the oviduct region: transcripts for nuclear receptors (PGR) were more abundant in the isthmus while those for progesterone membrane component 2 (PGRMC2) were higher in the ampulla in cattle^10^. Furthermore, it was shown in gilts that each oviductal region responded differently to estradiol, leading to different regional patterns of secretion^43^. Taken together, the observed region-specific profiling of the OF proteome could result from differences in blood flow, proportion of secretory cells, gene expression and steroid hormone receptivity between ampulla and isthmus.

After mating or insemination, sperm cross the uterus and reach the isthmus where they interact with both OECs and oviduct secretions; these interactions are believed to create an optimal environment for sperm storage until ovulation^2,44^. Of the BP/MF overrepresented exclusively at Pre-ov, proteins involved in cell redox homeostasis (including PRDX3, PRDX4, PDIA3, PDIA6 among others), negative regulation of apoptotic process (incl. HSPA5, HSP90B1, RGN) and microtubule motor activity (incl. DNAH1, KLC3, KIF21A) may be considered as possible candidates to provide an optimal environment for sperm survival and motility until fertilization. Of those protein candidates, HSP90B1, HSPA5 and PDIA3 were among the most abundant proteins in the isthmus (NWS ≥ 100) and were also 2 to 3 times more abundant than in the ampulla at Pre-ov. HSP90B1, PDIA3 and HSPA5 were previously identified as sperm-binding proteins in the OF in cattle^45^. In humans, the inhibition of PDIA3 by specific antibodies reduced sperm ability to bind to the zona pellucida^46^. In addition, both HSP90B1 and HSPA5 promoted sperm-zona binding in human^47^ and pigs^48^. Recombinant HSPA5 enhanced human sperm calcium influx, one step of sperm capacitation^49^. Calreticulin (CALR) was another protein found 2.5 times more abundant in the isthmus compared to the ampulla at Pre-ov. Calreticulin is a calcium-binding protein required for cumulus oocyte complex development^50^. Moreover, addition of recombinant CALR in the fertilization medium blocked polyspermy in pigs^51^.

After ovulation, sperm undergo capacitation and migrate gradually toward the ampulla where fertilization takes place, then embryo development starts in the isthmus. Hence, we focused on identified proteins that could play a key role in supporting fertilization, embryo transport and development. Numerous DAPs between regions (incl. HSPA5, HSP90B1, ANXA3, ANXA6, MYO1D, MYO1B, PHKB) were involved in calcium homeostasis, including calcium ion binding and calcium-dependent protein binding. Calcium is well known to play major roles in sperm functions such as motility, capacitation and acrosome reaction^52^. In addition, several proteins (GNAS, GNG2, GNB1) were involved in cell response to catecholamines. Catecholamines have been implicated in the spontaneous contractions of the bovine oviduct^53^ and may help embryo transport towards the isthmus. Among the overabundant proteins in the ampulla at Post-ov, oviductin (OVGP1) was among the most abundant proteins (NWS > 150) and 2.7 times more abundant than in the isthmus. Similarly, OVGP1 transcripts were more abundant in the ampulla than in the isthmus at day 4 post-ovulation in cows^12^. OVGP1 is an oviduct-specific glycoprotein secreted around the time of ovulation that plays major roles in zona pellucida hardening and control of polyspermy in mammals^54,55^. Addition of recombinant OVGP1 in a fertilization medium improved sperm-zona pellucida binding in hamster^56^ and humans^57^. Moreover, purified or recombinant OVGP1 positively influenced sperm viability, motility and capacitation in buffaloes^58^ and humans^57^. Furthermore, numerous proteins overabundant in the ampulla were involved in the immune system (incl. CFH, CFD, PLAT, PLTP). Complement factor H (CFH) acts as a soluble inhibitor of complement and plays as such an essential role in maintaining a well-balanced immune response. CFH was reported in boar seminal plasma and it was shown that sperm possessing CFH in their outer acrosomal region evaded complement attack in the female tract^59^. The mRNAs for phospholipid transfer protein (PLTP) were reported in the oviduct epithelium of mice and increased in abundance in the presence of embryos but not in pseudo-pregnant mice, suggesting important roles during early development^60^.

Beyond the oviduct region, the proximity of the ovulating ovary was another factor correlated with OF protein abundance. The regulatory effect of the POF/CL on OF proteome around the time of ovulation was previously described in cattle^9^ and horse^61^. Differentially expressed genes between sides were also identified in the ampulla and isthmus on day 3 post-estrus in cattle^62^. The proportion of secretory cells was reported to be equivalent between ipsilateral and contralateral oviducts^63^. However, highly asymmetrical concentrations of sex steroid hormones were reported in the bovine oviduct, with up to 3.5 times more estradiol before ovulation^64^ and up to 16 times more progesterone after ovulation^7^ in the ipsilateral oviduct than in the contralateral one. An asymmetry in the local vascularization and anatomy of the oviduct were also reported, with a thicker, more edematous and more transparent wall in the ipsilateral side^65^. Overall, those differences in monovular species could probably be involved in the asymmetrical regulation of oviduct secretions.

The POF/CL effect was expected to be an important regulatory factor, preparing the suitable environment for early embryo development. In accordance, the Post-ov isthmus was the most affected by the proximity of the POF/CL, with 248 DAPs compared with 83–177 DAPs in other pair-wise comparisons between sides of ovulation. In line with the present data, transcriptomic analysis of bovine OECs identified 13 times more differentially expressed genes between sides of ovulation in the isthmus than in the ampulla on Day 3 post-estrus^62^. In a previous proteomic study in cattle, the proportion of DAPs in the entire oviduct was also higher at Post-ov than at Pre-ov^9^. The signaling pathways most significantly altered by the proximity of ovulating ovary included the EGFR, ERBB2 and PI3-kinase (PI3K) pathways after ovulation. Recent results in dogs show that exosomes secreted by oviduct cells mediate the EGFR/mitogen-activated protein kinase (MAPK) signaling pathway, which is one of the prerequisite pathways for further development, in cumulus cells^66^. The PI3K pathway plays also pivotal roles in numerous reproductive processes^67^. The PI3K-Akt pathway was one of the most affected pathways in the oviduct after mating, as shown in a transcriptomic study in pigs^68^.

Studies on the factors correlated with differential protein abundance close to the ovulation time are still scarce because most studies compared the estrus/follicular phase to the luteal phase, with a time interval of around 5–12 days between conditions^11,37,69^. The time relative to ovulation (Pre-ov vs. Post-ov) was the factor with least variation between samples with only 2% of DAPs identified in the ampulla and isthmus. Higher rates of DAPs (13–17%) were previously evidenced in the bovine OF between stages but with no region separation^9^. Recently, differences before and after ovulation were reported in the equine OF proteome^36^. These authors reported 15 proteins downregulated in post-ovulatory OF compared to the pre-ovulatory counterpart, while 156 upregulated in post-ovulatory OF compared to pre-ovulatory OF. It is to note that the Pre-ov and Post-ov stages covered approximately 3–4 days around ovulation time and represented a limited time window relative to the 21 to 25-day length of the estrous cycle, which may explain the limited number of DAPs. Functions such as regulation of cilium assembly, intraciliary transport and negative regulation of cell proliferation were affected by the peri-ovulatory stage in the ampulla. The proportions of ciliated cells and of proliferative cells in the oviduct epithelium was reported to change during the estrous cycle in cows^39^ with an increase during the follicular phase and a decrease in the luteal phase, which may account for this stage-specific enrichment.

One limitation of the present study is the lack of information on cows’ breed, age and metabolic status at the time of OF collection since the samples were collected at a slaughterhouse. The OF proteome was obtained from a total of 32 animals, providing data representative for this species, yet, the samples were analyzed in pools of 4 animals, precluding any evaluation of individual variations.

In conclusion, this study provides a comprehensive characterization of the bovine OF proteome, revealing a fine-tune regulation of the OF according to the anatomical region of the oviduct, the proximity to the ovary and the stage of the cycle. The strong modulatory effect of the oviduct regions points out the anatomical region as the major regulator of OF proteome for the first time. Moreover, the in deep detail of the spatiotemporal proteomic changes of the OF around the ovulation window, enhance our understanding of the oviductal milieu supporting the early reproductive events. The proteins identified here paved the way for future functional experiments towards improving sperm and embryo biotechnologies.

## Methods

### Collection of bovine oviductal fluid (OF)

Both oviducts from adult cyclic cows were collected at a local slaughterhouse (40 min from the lab), placed immediately on ice and transported to the laboratory within max 2 h after animal death. The oviducts were classified into pre-ovulatory (Pre-ov; presence of a *corpus albicans* and one pre-ovulatory follicle of 11–20 mm in diameter, corresponding to approximately days 19–21 of cycle) or post-ovulatory (Post-ov; presence of an early *corpus luteum* made with red loosely organized tissue less than 1 cm in diameter, corresponding to approximately day 1–4 of cycle), as previously described^70^. Oviducts ipsilateral and contralateral to ovulation side were trimmed free of surrounding tissue and vessels and linearized. After removal of the infundibulum and utero-tubal junction, both oviducts were divided into two parts at the ampullary-isthmic junction. The ampulla and isthmus were individually flushed with 200 µL of cold sterile protein-free phosphate buffered saline (PBS) and kept on ice. Cellular debris and cells were eliminated from the OF by two centrifugations (2000*g* for 10 min then 12000*g* for 10 min) at 4 °C. Samples were stored at − 80 °C until proteomic analysis. A total of 18 Pre-ov and 16 Post-ov cows were collected. In the Pre-ov group, in order to exclude animals with ovarian cyst or atretic follicle, the follicular fluid of the Pre-ov follicle was collected and stored at − 80 °C for steroid hormone analysis, as previously described^71^. Concentrations of P4 were measured by a competitive enzyme-linked immunosorbent assay according to a method previously described^72^ whereas E2 concentrations were measured using the BioSource E2-EASIA kit (BioSource Europe S.A., Louvain-la-Neuve, Belgium). Assays were performed in duplicate. Two cows were excluded from the Pre-ov group based on intra-follicular P4 concentrations higher than 160 ng/mL, as previously described^7^. In the resulting Pre-ov group (n = 16), the ranges of P4 and E2 intra-follicular concentrations were 19.4–134.2 ng/mL and 103 to > 2000 ng/mL, respectively.

### Preparation of OF samples for proteomic analyses

The OF samples were thawed on ice and pools of 100 µL of OF were made by mixing 25 µL of individual samples. Four pools of 4 cows per side × region × stage condition were constituted, leading to 32 OF samples distributed in 8 conditions (Pre-ov ipsilateral ampulla; Pre-ov ipsilateral isthmus; Pre-ov contralateral ampulla; Pre-ov contralateral isthmus; Post-ov ipsilateral ampulla; Post-ov ipsilateral isthmus; Post-ov contralateral ampulla and Post-ov contralateral isthmus). In the following, the term “sample” refers to these pools of OF. Protein concentration of OF samples was assessed using the Uptima BC Assay kit (Interchim, Montluçon, France) according to manufacturer’s instructions and using bovine serum albumin as a standard. The homogeneity and quality of samples was checked after migration of the OF pools (5 μg proteins per lane) on a 4–20% SDS-PAGE (80 V, 10 min and 180 V 30 min) and staining with Coomassie blue (see Supplementary Fig. S3).

### Nanoliquid chromatography coupled with tandem mass spectrometry (NanoLC-MS/MS)

For proteomic analysis, 10 µg of proteins per sample were prepared using the PreOmics iST-BCT kit (PreOmics GmbH, Martinsried, Germany) following the manufacturer’s instructions. Briefly, proteins were lysed, denatured, reduced and alkylated for 10 min at 95° then Trypsin/LysC digested for 60 min at 37 °C. Purification and elution of peptides were then carried out for 1 h at room temperature. Samples were then purified from salts, contaminants and other detergents, using reversed phase SDBS spin cartridge (PreOmics GmbH, Martinsried, Germany). Peptide injection and analysis were performed as previously described^32^. The resulting peptide mixtures were loaded on a 75 µm × 250 mm IonOpticks Aurora 2 C18 column (Ion Opticks Pty Ltd., Bundoora, Australia). A gradient of basic reversed-phase buffers (Buffer A: 0.1% formic acid, 98% H_2_O MilliQ, 2% acetonitrile; Buffer B: 0.1% formic acid, 100% acetonitrile) was run on a NanoElute HPLC System (Bruker Daltonik GmbH, Bremen, Germany) at a flow rate of 400 nL/min at 50 °C. The liquid chromatography (LC) run lasted for 120 min (2% to 15% of buffer B during 60 min; up to 25% at 90 min; up to 37% at 100 min; up to 95% at 110 min and finally 95% for 10 min to wash the column). The column was coupled online to a TIMS TOF Pro (Bruker Daltonik GmbH, Bremen, Germany) with a CaptiveSpray ion source (Bruker Daltonik). The temperature of the ion transfer capillary was set at 180 °C. Ions were accumulated for 114 ms, and mobility separation was achieved by ramping the entrance potential from − 160 to − 20 V within 114 ms. The acquisition of the MS and MS/MS mass spectra was done with average resolutions of 60,000 and 50,000 full width at half maximum (mass range 100–1700 m/z), respectively. To enable the PASEF method, precursor m/z and mobility information was first derived from full scan TIMS-MS experiments (with a mass range of m/z 100–1700). The quadrupole isolation width was set to 2 and 3 Th and, for fragmentation, the collision energies varied between 31 and 52 eV depending on the precursor mass and charge. TIMS, MS operation and PASEF were controlled and synchronized using the control instrument software OtofControl 6.2 (Bruker Daltonik). LC–MS/MS data were acquired using the PASEF method with a total cycle time of 1.31 s, including 1 TIMS MS scan and 10 PASEF MS/MS scans. The 10 PASEF scans (100 ms each) containing, on average, 12 MS/MS scans per PASEF scan. Ion mobility-resolved mass spectra, nested ion mobility vs. m/z distributions, as well as summed fragment ion intensities were extracted from the raw data file with DataAnalysis 5.3 (Bruker Daltonik GmbH, Bremen, Germany). Signal-to-noise (S/N) ratio were increased by summations of individual TIMS scans. Mobility peak positions and peak half-widths were determined based on extracted ion mobilograms (± 0.05 Da) using the peak detection algorithm implemented in the DataAnalysis software. Features detection were also performed using DataAnalysis 5.3 software and exported in .mgf format.

### Protein identification and data validation

Peptides were identified using the MASCOT software (version 2.5.1; Matrix Science, London, UK) against the Uniprot *Bos taurus* database (May 2019, 23,523 sequences) using its automatic decoy database search to calculate a false discovery rate (FDR). The parameters used for database searches included trypsin as enzyme (one missed cleavage allowed), carbamidomethylcysteine as fixed modification, oxidation of methionine and N-terminal protein acetylation as variable modifications. Monoisotopic mass was considered and mass tolerance was set at 15 ppm for MS ions and 0.05 Da for MS/MS ions. Mascot results from the target and decoy databases were incorporated to Scaffold Q + software (version 5.0.1, Proteome Software, Portland, USA, http://www.proteomesoftware.com). Threshold for peptide and protein identification were set to 95.0% as specified by the Peptide Prophet algorithm^73^ and the Protein Prophet algorithm^74^.

### Label-free protein quantification and statistical analysis

All proteins containing at least two unique peptides (False discovery rate (FDR) < 0.01%) were considered for protein quantification. The abundance of proteins was assessed using the Spectrum Count quantitative method and the “weighted spectra” option of the Scaffold software, as previously described^32^. The weighted spectra option of Scaffold is based on the assignment of each peptide to a weight according to whether they are shared or not between identified proteins. The normalization of data was achieved by multiplying each spectrum count in each sample by the average count over the sample’s total spectrum count.

Statistical analysis was assessed on proteins with on average 2 normalized weighted spectra (NWS) or more in at least one condition. To obtain an overview of proteomic data, principal component analysis (PCA) of all samples was carried out using the FactoMineR and ggplot2 packages of RStudio software (version 1.4.1106). Analysis of variance on biological replicates and a heatmap representation of differentially abundant proteins (DAPs; p-value ≤ 0.05) were carried out using the FactoMineR and gplot packages of RStudio. Pair-wise comparison between stage × region × side conditions were evaluated by Student *t*-tests using the Scaffold software. Proteins were considered as differentially abundant with a t-test p-value ≤ 0.05 and a min fold-change ratio of 2. Lists of top-20 differentially abundant proteins are the quantitatively major proteins (based on NWS) at one given stage, side or region.

### Prediction of secretory pathways and functional enrichment analysis

Identification of signal peptide-containing proteins and prediction of unconventional protein secretion (UPS) was first carried out using the Outcyte 1.0 online tool (http://www.outcyte.com/). The list of gene names was used as input and the default score threshold of 0.5 was considered for UPS prediction. Proteins having a transmembrane domain and predicted to be intracellular were also predicted by this software. The identification of signal peptide sequences was then checked on all proteins using the SignalP online tool (version 5.0; http://www.cbs.dtu.dk/services/SignalP/). Proteins identified were also compared to those previously reported in OF-derived EVs in cattle^19^, cats^20^ and to OEC-derived EVs in pigs^21^. The gene enrichment analysis was performed separately for each pairwise comparison except for region and stage effects for which the analysis was restricted to the ipsilateral side of ovulation. The gene lists of DAPs were first imported in the Database for Annotation, Visualization and Integrated Discovery (DAVID version 6.8) for gene ontology (GO) analysis. Overrepresented molecular functions (MF) and biological processes (BP) GO terms with a p-value < 0.05 were considered as significant. The KEGG pathways associated with DAPs were further analyzed using the Proteomaps software (http://bionic-vis.biologie.uni-greifswald.de/). Proteomaps graphics were generated from NWS values of overabundant DAPs in each pair-wise comparison. To evaluate the potential roles of DAPs in oviductal events, the Metascape Membership analysis tool was used (https://metascape.org/)^22^. Enrichment analysis of DAPs matching the membership terms “sperm”, “oocyte”, “ovulation”, “fertilization” and “embryo” were retained. The *Homo sapiens* genome was used as background dataset for all analyses as it is better annotated than the *Bos taurus* genome.

## Supplementary Information

Supplementary Information.

Supplementary Data S1.

Supplementary Data S2.

Supplementary Data S3.

Supplementary Data S4.

## Data Availability

The mass spectrometry data have been deposited to the ProteomeXchange Consortium via PRIDE (https://www.ebi.ac.uk) with the dataset identifier PXD030915 and 10.6019/PXD030915
