# Supplementary material for: Spatiotemporal profiling of the bovine oviduct fluid proteome around the time of ovulation
Source: Sci Rep. 2022 Mar 9;12:4135. doi: 10.1038/s41598-022-07929-3 (PMC8907256; doi:10.1038/s41598-022-07929-3)
Supplement: Supplementary file 1 — Supplementary Information. [file 41598_2022_7929_MOESM1_ESM.pdf]

Supplementary information

Spatiotemporal profiling of the bovine oviduct fluid proteome around the time of ovulation

Coline Mahé[1*], Régis Lavigne[2,3], Emmanuelle Com[2,3], Charles Pineau[2,3], Yann Locatelli[1,4], Aleksandra Maria Zlotkowska[5,#], Carmen Almiñana[1,6], Guillaume Tsikis[1], Pascal Mermillod[1], Jennifer Schoen[5,#], Marie Saint-Dizier[1,7]

[1] CNRS, IFCE, INRAE, Université de Tours, PRC, 37380, Nouzilly, France

[2] Univ Rennes, Inserm, EHESP, Irset (Institut de recherche en santé, environnement et travail) - UMR-S 1085, F-35000 Rennes, France; emmanuelle.com@univ-rennes1.fr (E.C.); regis.lavigne@univ-rennes1.fr (R.L.); charles.pineau@inserm.fr (C.P.)

[3] Protim, Univ Rennes, Biosit – UMS 3480 CNRS, US 018 Inserm, F-35000 Rennes, France

[4] MNHN, Laboratoire de la Réserve Zoologique de la Haute Touche, Obterre, France

[5] Institute of Reproductive Biology, Leibniz Institute for Farm Animal Biology, FBN, Dummerstorf, Germany

[6] Functional Genomics Group, Institute of Veterinary Anatomy, Vetsuisse Faculty Zurich, University of Zurich, 8315 Lindau, Switzerland

[7]Tours University, Faculty of Sciences and Techniques, Tours, France

#Current address: Department of Reproduction Biology, Leibniz Institute for Zoo and Wildlife Research (IZW), Berlin, Germany

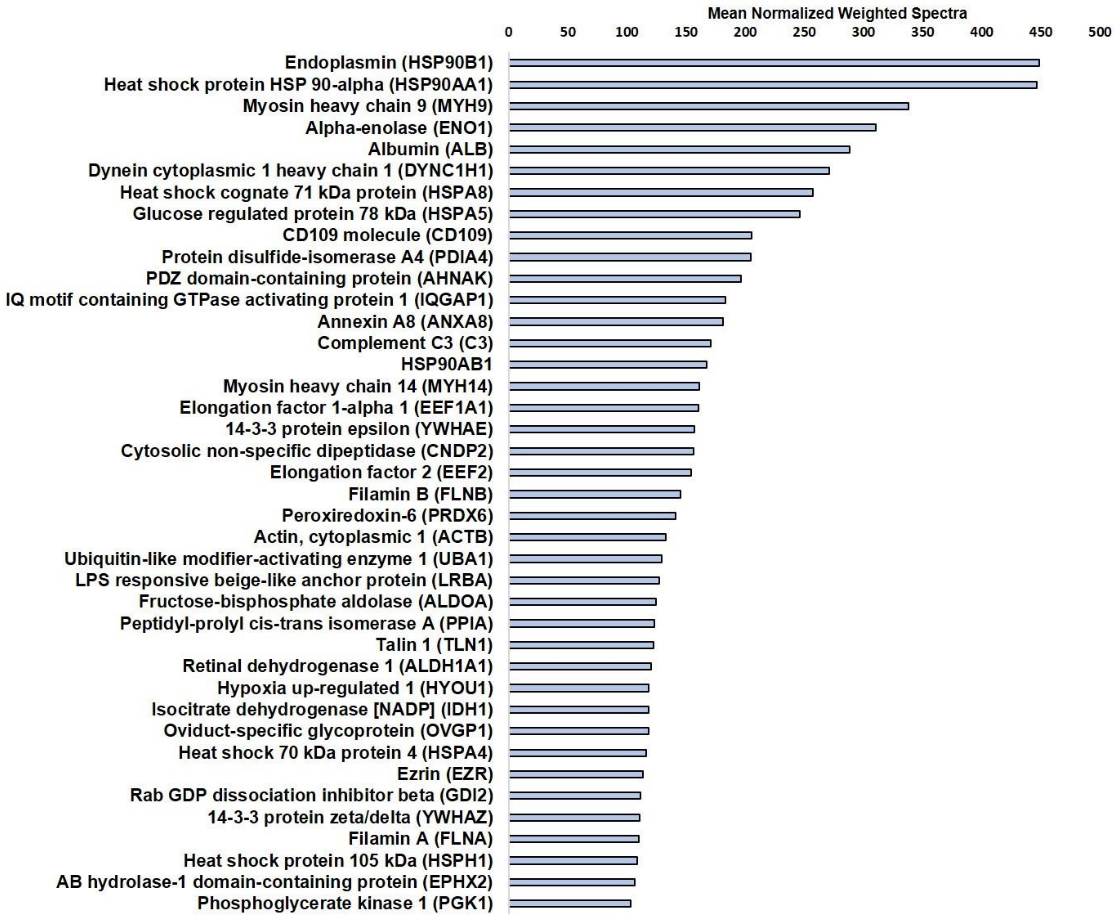

Supplementary Figure 1. Mean quantitative values of the top-40 most abundant proteins in all samples.

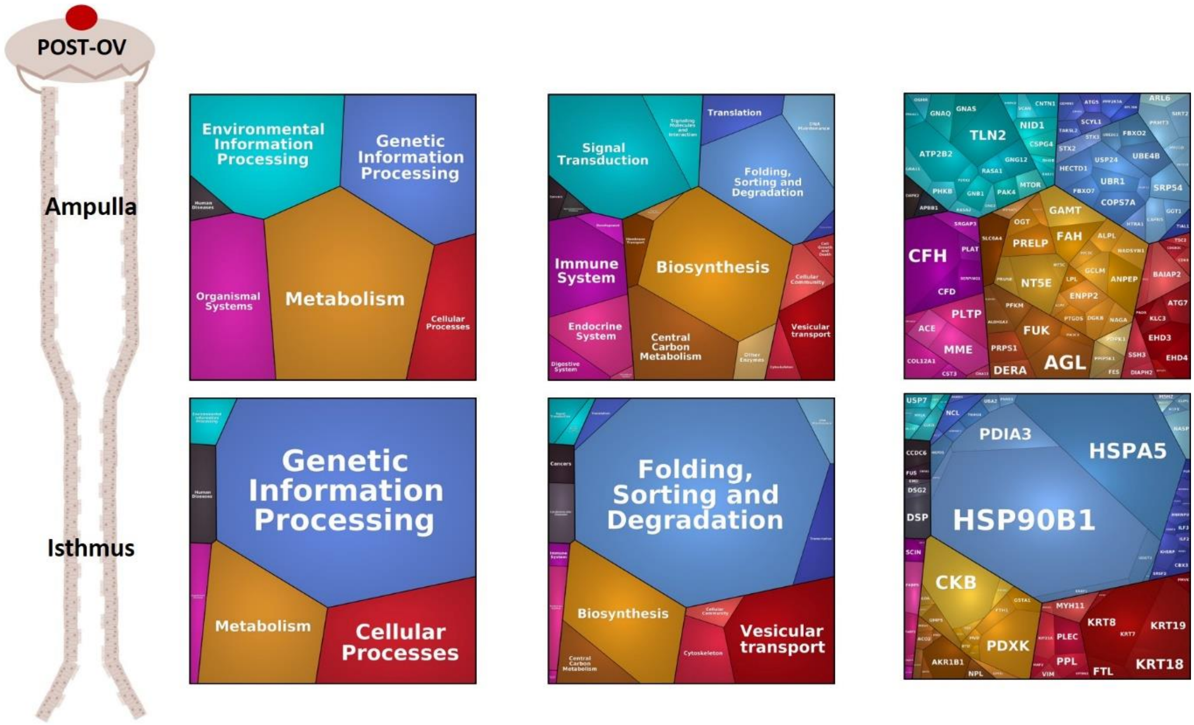

Supplementary Figure 2. Proteomaps of overabundant proteins in the ipsilateral ampulla and isthmus at Post-ov stage. Proteomaps were generated using the KEGG (Kyoto Encyclopedia of Genes and Genomes) Pathway gene classification. Functional categories (left and middle panels) and proteins (right panels) are shown by polygons. Areas of polygons illustrate protein normalized abundance in each region. Functions and proteins linked are organized in common regions and coded using similar colors.

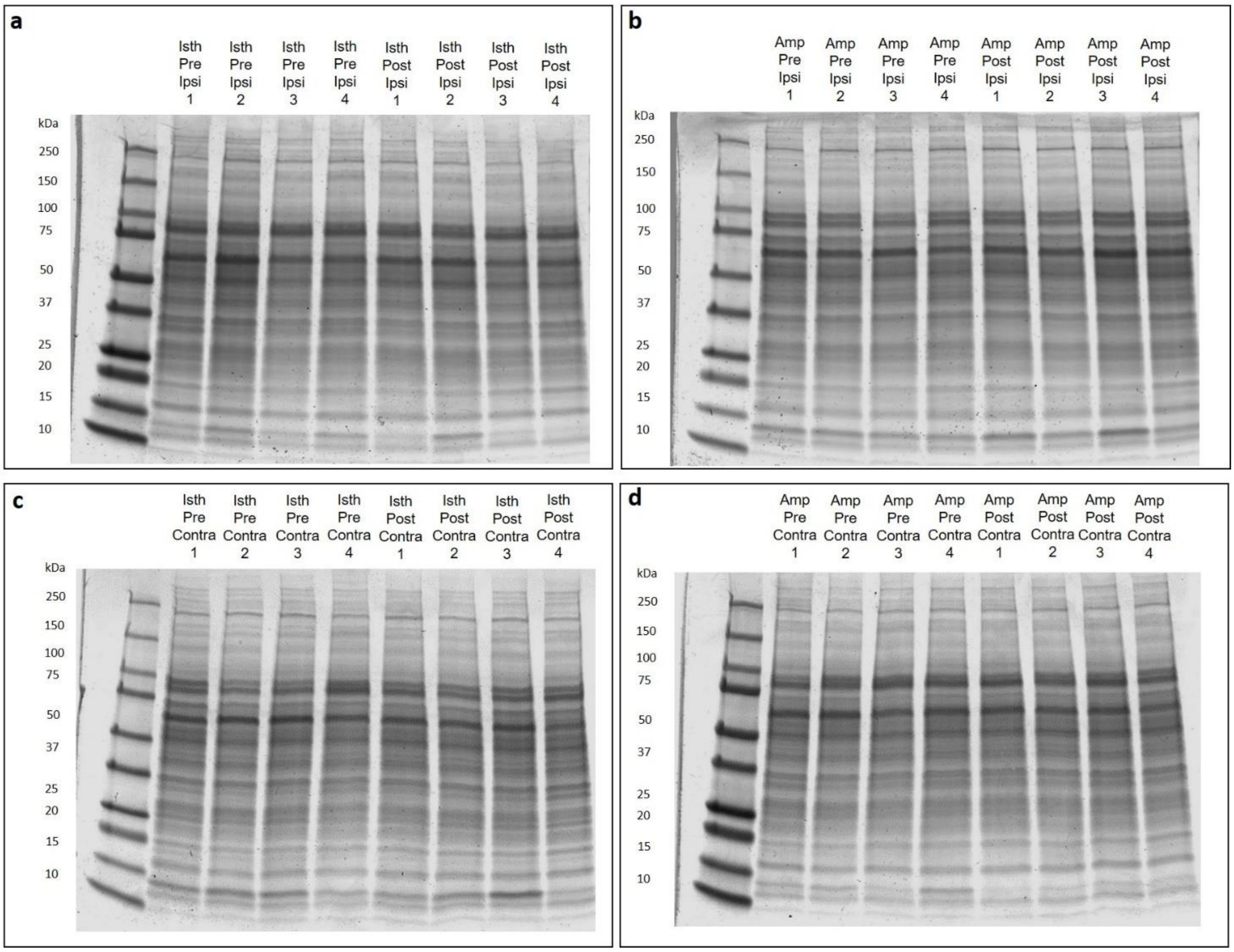

Supplementary Figure 3. Protein profile of the 32 oviductal fluid samples. 5 µg of OF at both stages from (a) the ipsilateral isthmus (b) the ipsilateral ampulla (c) the contralateral isthmus and (d) the contralateral ampulla were migrated on a 4-20% SDS-page and stained with Coomassie blue.

Supplementary Dataset File 1. List of proteins identified in the bovine oviductal fluid and predicted to be intracellular, secreted and in oviduct extracellular vesicles. Prediction of secretory pathways were carried out using the tools online Outcyte and SignalP 5.0**.**

Supplementary Dataset File 2. List and functional analysis of differentially abundant proteins between regions at peri-ovulatory stages in the bovine OF ipsilateral to

ovulation. Gene ontology terms from DAVID analysis are ordered according to their significance**.**

Supplementary Dataset File 3. List and functional analysis of differentially abundant proteins between ovulatory sides in the bovine OF. Gene ontology terms from DAVID analysis are ordered according to their significance**.**

Supplementary Dataset File 4. List and functional analysis of differentially abundant proteins between peri-ovulatory stages in the bovine OF ipsilateral to ovulation. Gene ontology terms from DAVID analysis are ordered according to their significance**.**
